# Efficacy of non-specific hemostatic agents for reversal of prophylactic apixaban levels

**DOI:** 10.1186/2197-425X-3-S1-A910

**Published:** 2015-10-01

**Authors:** K Schmidt, K Krüger, E Langer, M Körber, C von Heymann, K-D Wernecke

**Affiliations:** Charité University Medicine Berlin, Anaesthesiology, Berlin, Germany; Labor Berlin GmbH, Laboratory, Berlin, Germany; Vivantes Klinikum im Friedrichshain, Anaesthesiology and Intensive Care, Berlin, Germany; Sostana GmbH, Statistical Analysis, Berlin, Germany

## Introduction

Apixaban (Eliquis^®^) is a direct and competitive inhibitor of factor FXa that is approved for thrombosis prophylaxis after hip and knee replacement surgery, in non-valvular atrial fibrillation and venous thromboembolic events therapy [1]. In cases of severe hemorrhages there is no approved specific antidote available to reverse the effect of apixaban yet. Previous animal and in vitro studies [2, 3] with supratherapeutic concentrations of apixaban (200ng ml^-1^) have shown that activated prothrombin complex concentrate (aPCC) and recombinant factor VIIa (rFVIIa) have a greater effect in reversing the effect of apixaban than prothrombin complex concentrate (PCC). The effect of these non-specific hemostatic agents for reversal of apixaban concentrations measured in patients after prophylactic doses (maximum observed plasma concentration 62 ng ml^-1^ [4]) remains unclear.

## Objectives

Evaluation of the efficacy of PCC, aPCC and rFVIIa for reversal of prophylactic concentration of apixaban induced alterations in hemostasis.

## Methods

Blood samples from 10 healthy volunteers were spiked with apixaban in a corresponding dose of 2.5 mg twice daily (4) and clinically relevant concentrations of PCC: Cofact^®^: 25 IU kg^-1^ (0,35 IU ml^-1^), aPCC: FEIBA^®^ 25 IU kg^-1^ (0,35 IU ml^-1^) and rFVIIa: Novoseven^®^ 90µg kg^-1^ (1 µg ml^-1^). Tests were performed including thromboelastometry, prothrombin time (PT) and activated partial thromboplastin (aPTT). Statistical analysis was performed using non-parametric Wilcoxon pair-wise test.

## Results

Apix-apixaban; Data are median (95% confidence interval). *p < 0,05 vs. Control, #p < 0,05 vs. apix

Prolongations in measured latency parameters were corrected by the different concentrates with variable efficacies (rFVIIa≥aPCC>PCC). Addition of aPCC and rFVIIa to the spiked blood samples leads to overcorrection of PT, aPTT and CT-EXTEM.

## Conclusions

Recombinant FVIIa and aPCC have the potential to restore the induced alterations in hemostasis of apixaban in prophylactic dose in vitro. PCC showed partial effects only. The reversal effects of activated factor concentrates tend to overcorrection, which might be a risk for thrombotic events.

## Grant Acknowledgment

Research Grant from Bristol-Myers Squibb/Pfizer.

**Table 1:** Table 1

	Control	apix	PCC 0,35IU ml-1	aPCC 0,35IU ml-1	rFVIIa 1µg ml-1
**apix ng ml-1**	0	53	55	53	53
**CT-EXTEM (s)**	70	78	78#	57*#	52*#
**aPTT (s)**	34,6	37,0*	37,3*#	32,9*#	29,5*#
**PT (s)**	13,7	14,0*	13,0*#	11,5*#	9,0*#
